# Factors Influencing Cervical Cancer Screening Uptake among Reproductive-Aged Filipino Women: Findings from the 2022 Philippines National Demographic and Health Survey

**DOI:** 10.1089/whr.2024.0011

**Published:** 2024-06-05

**Authors:** Wah Wah Myint, Roaa Aggad, Qiping Fan, Sara E. Mendez

**Affiliations:** ^1^ Department of Health Behavior, Center for Community Health and Aging, School of Public Health, Texas A&M University, College Station, Texas, USA.; ^2^ Department of Family and Community Medicine, King Abdulaziz University, Rabigh, Saudi Arabia.; ^3^ Department of Public Health Sciences, Clemson University, Clemson, South Carolina, USA.

**Keywords:** cervical cancer screening, intimate partner violence, social determinants, the Philippines

## Abstract

**Background:**

Cervical cancer (CC) is the fourth leading cause of death among cancer cases and women intimate partner violence (IPV) survivors are more likely to experience CC-related mortality. This study aims to evaluate the factors influencing CC screening uptake among reproductive-aged women, especially among IPV survivors in the Philippines.

**Method:**

We used the 2022 Philippines’ National Demographic and Health Survey. The outcome variable was undergoing CC screening. The independent variables were different types of IPV, sociodemographic characteristics (age groups, place of residency, education level, wealth quintile, marital status, religion, employment), and other important variables (number of sexual partners, number of children, and access to health care). Descriptive statistics and multivariable logistic regression analyses were performed to examine influencing factors of CC screening.

**Method:**

The results revealed that approximately 10% (*n* = 1,648) of the women who participated in the survey had screened for CC. The results showed that women who experienced at least one type of IPV (adjusted odds ratio [aOR] = 1.32, 95% confidence interval [CI] = 1.08–1.62), aged 45–49 years (aOR = 6.42, 95% CI = 2.60–15.54), higher education (aOR = 14.26, 95% CI = 3.28–61.99), wealthier (aOR = 3.46, 95% CI = 2.54–4.72), having current employment (aOR = 1.30, 95% CI = 1.08–1.57), and having more than five lifetime sexual partners (aOR = 3.16, 95% CI =1.00–9.97), were more likely to undergo CC screening than their counterparts.

**Conclusion:**

Future CC screening initiatives should prioritize women with lower educational and socioeconomic backgrounds to effectively bridge the gaps in health disparities.

## Introduction

Cervical cancer (CC) is the second most prevalent cancer globally, and the fourth most common cancer among women with estimated 604,000 CC cases detected in 2020.^1–4^ Globally, 341,831 CC mortality occurred in 2020.^1–3,5–7^ With this rate, half a million women will likely die of CC annually by 2030 and yet, many women still face barriers to receive access to CC screening.^8–10^ CC is the third most typical cancer in developing countries, particularly in Africa (*e.g.,* Kenya),^11^ and second-most in Southeast Asia (*e.g.,* the Philippines) where the prevalence has been exacerbated by a lack of screening and sufficient treatment services.^2,12–19^ A study conducted by Hung and colleagues revealed that CC mortality in the Philippines has significantly increased from 1980 to 2018.^20^ Education, vaccination, screening and early detection, and prompt treatment have been shown to effectively reduce the incidence rates of CC.^21–30^ According to Bruni and colleagues, about 39.6 million Filipino women aged 15 years and older were at risk for CC in 2020, and it is estimated that 7,897 new CC cases are diagnosed annually.^19^ Although the HPV vaccine is available for Filipino women, its coverage in 2020 in the Philippines was only 23% for the first dose and 5% for the completed dose.^31,32^ In addition, beginning in 2022, the Philippines started an active CC screening program although official national recommendations for the screening for women aged 25 to 55 years are present.^32,33^ The current scientific evidence showed that HPV vaccination has protective factor for CC for more than 10 years if administered early. The World Health Organization (WHO) set global targets of “90–70–90” for countries, including the Philippines, to eliminate CC by 2030.^34^ This means that 90% of girls receive a human papillomavirus (HPV) vaccination by age 15, 70% of women are screened with a high-quality test by ages 35–45 years, and 90% of women with cervical disease receive treatment.^34^ However, the Philippines data showed that numerous efforts are needed to reach the 90–70–90 targets by 2030.^34^ With the current gaps in screening services, women with CC could get late diagnoses, leading to increasing burden for them, their families, and the health system. Therefore, it is necessary to advance stringent guidelines for women to undertake screening for CC.^34^

Intimate partner violence (IPV) has been one of the major public health problems impacting millions of people around the world.^35,36^ IPV victimizations are estimated at 7.7 million annually.^37^ Approximately one in three women aged 15–49 years experienced at least one form of IPV, including physical, sexual, or emotional abuse.^36^ Women were disproportionally impacted by IPV, and such traumatic experiences may lead to negative health consequences, including anxiety, depression, sexually transmitted infections, and other chronic conditions.^36^ Many studies have found that IPV is linked to CC.^37^ Women who experienced IPV are more predisposed to CC compared with those who did not.^35,38^ There were variations in CC rates depending on a woman’s history of exposure to violence, its duration, severity, and the victim’s age at the time of exposure. Whether it is emotional, physical, or sexual violence, women often suffer trauma that has a lasting impact.^35^

The intersection of IPV and cancer diagnosis, including CC, has created a novel population of women in need.^35^ Although many studies have found a link between CC and IPV, there is limited literature studying the association between CC screening, specifically among women who have experienced IPV. Undergoing screening is problematic for most low-income women,^39,40^ especially IPV women who are highly unlikely to receive preventive health care services.^35^ Such hesitancy is attributed to trauma associated with violence.^41^ Barriers to screening and delayed treatment result in CC’s high incidence at a later stage.^36^ Hence, it is necessary to undertake comprehensive research on this topic to add to the evidence for future public health interventions and policies.

Our study aims to identify the factors that influence CC screening in women, particularly those experiencing IPV. The objective is to examine the encouraging and discouraging factors of women seeking CC screening services. The study’s findings will add to the program designs that augment CC awareness and the prevention of IPV, allowing policymakers to take immediate action to achieve the universal targets by 2030.

## Materials and Methods

The Philippines Statistics Authority implemented the Philippines’ National Demographic and Health Survey (NDHS) with the funding and technical support from the United States Agency for International Development.^42^ The NDHS survey was reviewed and approved by the Institutional Review Board of the Inner-City Fund.^42^ All participants provided verbal consent to participate in the survey.^42^ The NDHS study applied a two-stage probability sampling design to have a representative sample.^42^ We analyzed data (*n* = 27,821) from the NDHS conducted in the Philippines in 2022. (42)^43,44^ Of the total number of respondents, 19,228 women completed the domestic violence module of the survey and were included in the present study.

### Measures

The outcome variable used in this study was “Ever had cervical cancer screening,” which is a binary variable (no = never screened, yes = had CC screening). The exposure variables were experience of different types of IPV, which is a dummy variable (no = no IPV, yes = at least one type of IPV). The covariates included sociodemographic characteristics, such as age in 5-year groups (15–19, 20–24, 25–29, 30–34, 35–39, 40–44, and 45–49 years), place of residence (urban, rural), respondent’s education level (no education, primary, secondary, higher), wealth quintile (poorest, poorer, middle, richer, richest), marital status (never in union, currently in union/living with a man, formerly in union/living with a man), religion (other religions, roman catholic), and respondents’ current employment status (no, yes). Other relevant covariates include number of lifetime sexual partners (only one, two to five, more than five), parity (no child, only one child, two to five children, more than five children), and access to health care (Not a big problem, a big problem). The variable access to health care was created from the four variables: are there any problems with permission to go, getting money, distance to health facility, and not wanting to go alone. Table 1 presents detailed descriptions of all the variables used in this study.

**Table 1.** Nature and Type of Included Variables

Variables	Responses	Nature
Outcome variable		
Ever had cervical cancer screening	No, Yes	Dummy
Exposure variable		
Experienced at least one type of intimate partner violence (IPV)	No, Yes	Dummy
Emotional violence	No, Yes	Dummy
Sexual violence	No, Yes	Dummy
Physical violence	No, Yes	Dummy
Sociodemographic characteristics		
Age groups	15–19, 20–24, 25–29, 30–34, 35–39, 40–44, 45–49	Categorical
Place of residency	Urban, Rural	Dummy
Education level	No education, Primary education, Secondary, Higher	Categorical
Wealth quintile	Poorest, Poorer, Middle, Richer, Richest	Categorical
Marital status	Never in union, Currently in union/living with a man, Formerly in union/living with a man	
Religion	Others^*^, Roman Catholic	Dummy
Current employment	No, Yes	Dummy
Number of lifetime sexual partner	Only one, Two to five, More than five	Categorical
Parity	No child, Only one, Two to five children, More than five children	Categorical
Access to health care	Not a big problem, A big problem	Dummy

Note.

^*^ *Religion*: Others include Protestant, Iglesia ni Cristo, Aglipay, Islam, other Christian, no religion.

*Physical violence*: Pushed, shook, or threw something at her; slapped, twisted her arm or pulled her hair; punched with his fist or with something that could hurt; Kicked, dragged her, or beat her up; tried to choke her or burn on purpose; attacked her with a knife, gun, or other weapon.

*Sexual violence:* Physically forced her to have sexual intercourse with him when the woman did not want to, physically forced her to perform any other sexual acts she did not want to, forced her with threats or in any other way to perform sexual acts she did not want to.

*Emotional violence:* Said or did something to humiliate her in front of others; threatened to hurt or harm her or someone she cared about; insulted her or made her feel bad about herself; did not allow her to engage in any legitimate work or practice her profession; controlled her money or property or forced her to work; destroyed her personal property, pets, or belongings or threatened or harmed her pets; had other intimate relationships.

### Statistical analyses

We performed univariate analyses to present the prevalence of characteristics of the respondents. We conducted Pearson’s chi-squared test (χ2) to examine the association between the “ever had for cervical cancer screening” and the IPV variables, and other covariates. A series of multivariable logistic regression analyses were performed. In the multivariable logistic regressions, except the exposure variables (*i.e.,* different types of IPV), we included the variables that showed significant association in the bivariate analyses. We reported adjusted odds ratio (aOR) with 95% confidence intervals (CIs) and *p*-values. The survey weights were applied in all our analyses applying svy and syvset commands by using Stata 17.0.^43^

## Results

As shown in Table 2, more than 90% of the respondents have not undergone CC screening and almost 51% reported that access to the nearest health facility was a big problem. At least 17.5% of the respondents had one form of IPV (Table 2). The results from the bivariate analyses revealed that all the included covariates had significant association with the CC screening (Table 3). Among the different types of violence, the association between CC screening and emotional violence (*p* = 0.039), as well as at least one type of violence (*p* = 0.048), shows a significant association.

**Table 2.** Prevalence of Cervical Cancer Screening, Intimate Partner Violence (IPV), Sociodemographic Characteristics, and Other Important Covariates (Weighted)

	Frequency	Percent
Outcome variable		
Ever had cervical cancer screening		
No	17,580	90.31
Yes	1,648	9.69
Total	19,228	100
Exposure variables		
At least one type of IPV		
No	11,865	82.50
Yes	2,721	17.50
Total		100
Emotional violence		
No	12,218	84.80
Yes	2,368	15.20
Total	14,586	100
Sexual violence		
No	14,141	97.71
Yes	345	2.25
Total	14,586	100
Physical violence		
No	13,593	93.58
Yes	991	6.42
Total	14,586	100
Sociodemographic characteristics		
Age in 5-year groups		
15–19	3,021	19.13
20–24	2,776	16.79
25–29	2,890	13.71
30–34	3,121	13.58
35–39	2,738	12.58
40–44	2,512	12.64
45–49	2,170	11.58
Total	19,228	100
Place of residence		
Urban	7,743	56.44
Rural	11,485	43.56
Total	19,228	100
Education level		
No education	212	0.67
Primary	2,290	9.07
Secondary	9,622	52.66
Higher	7,104	37.60
Total	19,228	100
Wealth quintile		
Poorest	5,067	16.51
Poorer	4,231	18.71
Middle	3,561	20.42
Richer	3,194	21.71
Richest	3,175	22.65
Total	19,228	100
Marital status		
Never in union	6,322	40.94
Currently in union/living with a man	12,280	55.81
Formerly in union/living with a man	626	3.25
Total	19,228	100
Religion		
Other religions	5,730	23.55
Roman Catholic	13,498	76.45
Total	19,228	100
Current employment		
No	10,583	54.77
Yes	8,645	45.23
Total		100
Number of lifetime sexual partners		
Only one	10,846	74.38
Two to five	3,269	25.01
More than five	53	0.61
Total	14,168	100
Parity		
No child	6,813	42.95
Only one	3,025	14.80
Two to five	8,562	38.56
More than five	828	3.68
Total	19,228	100
Access to health care		
Not a big problem	9,060	49.10
A big problem	10,168	50.90
Total	19,228	100

**Table 3.** Results from Bivariate Analysis: Association between Cervical Cancer Screening and Covariates (Weighted)

	Outcome: Ever had cervical cancer screening			
	No: *N* (%)	Yes: *N* (%)	Total	*p*-value
Exposure variables				
At least one type of intimate partner violence				
No	10,653 (87.52)	1,212 (12.48)	11,856	0.048
Yes	2,373 (85.22)	348 (14.78)	2,721	
Total	13,026 (87.12)	1,560 (12.88)	14,586	
Emotional violence				
No	10,959 (87.51)	1,259 (12.49)	12,218	0.039
Yes	2,067 (84.94)	301 (15.06)	2,368	
Total	13,026 (87.12)	1,560 (12.88)	14,586	
Sexual violence				
No	12,732 (87.21)	15,09 (12.79)	14,241	0.129
Yes	294 (83.16)	51 (16.84)	345	
Total	13,026 (87.12)	1,560 (12.88)	14,586	
Physical				
No	12,171 (87.26)	1,424 (12.74)	14241	0.156
Yes	855 (85.04)	136 (14.96)	991	
Total	13,026 (87.12)	1,560 (12.88)	14,586	
Sociodemographic characteristics				
Age groups				
15–19	2,999 (99.00)	22 (1)	3,021	<0.001
20–24	2,706 (97.29)	70 (2.71)	2,776	
25–29	2,716 (91.92)	174 (8.08)	2,890	
30–34	2,784 (85.84)	337 (14.16)	3,121	
35–39	2,406 (86.24)	332 (13.76)	2,738	
40–44	2,134 (83.24)	378 (16.31)	2,512	
45–49	1,835 (80.24)	335 (19.76)	2,170	
Total	17,580 (90.31)	1,648 (9.69)	16,207	
Place of residence				
Urban	6,863 (88.54)	880 (11.46)	7,743	<0.001
Rural	10,717 (92.61)	768 (7.39)	11,485	
Total	17,580 (90.31)	1,648 (9.69)	19,228	
Education level				
No education	209 (99.45)	3 (0.55)	212	<0.001
Primary	2,148 (93.16)	142 (6.84)	2,290	
Secondary	8,999 (92.75)	623 (7.25)	9,622	
Higher	6,224 (86.05)	880 (13.95)	7,104	
Total	17,580 (90.31)	1,648 (9.69)	19,228	
Wealth quintile				
Poorest	4,844 (95.95)	223 (4.05)	5067	<0.001
Poorer	3,983 (93.58)	248 (6.42)	4,231	
Middle	3,243 (90.67)	318 (9.33)	3,561	
Richer	2,866 (89.66)	328 (10.34)	3,194	
Richest	2,644 (83.80)	531 (16.20)	3,175	
Total	17,580 (90.31)	1,648 (9.69)	19,228	
Marital status				
Never in union	6,154 (96.68)	168 (3.32)	6,322	
Currently in union/living with a man	10,880 (85.89)	1,400 (14.11)	12,280	<0.001
Formerly in union/living with a man	546 (86.02)	80 (13.98)	626	
Total	17,580 (90.31)	1,648 (9.69)	19,228	
Religion				
Other religions	5,366 (92)	346 (8)	5,730	0.012
Roman Catholic	12,214 (90)	1,284 (10)	13,498	
Total	17,580 (90.31)	1,648 (9.69)	19,228	
Current employment				
No	9,983 (93.95)	600 (6.05)	10,583	<0.001
Yes	7,597 (85.91)	1,048 (14.09)	8,645	
Total	17,580 (90.31)	1,648 (9.69)	19,228	
Number of lifetime sexual partners				
Only one	9,730 (87.58)	1,116 (12.42)	10,846	<0.001
Two to five	2,818 (82.98)	451 (17.02)	3,269	
More than five	42 (73.26)	11 (26.74)	53	
Total	12,590 (86.34)	1,578 (13.66)	14,168	
Parity				
No child	6,574 (96.08)	239 (3.29)	6,813	<0.001
Only one	2,698 (87.29)	327 (12.71)	3,025	
Two to five	7,551 (85.01)	1,011 (14.99)	8,562	
More than five	757 (90.75)	71 (9.25)	828	
Total	17,580 (90.31)	16,48 (9.69)	19,228	
Access to health care				
Not a big problem	8,202 (88.79)	858 (11.21)	9,060	<0.001
A big problem	9,378 (91.78)	790 (8.22)	10,168	
Total	17,580 (90.31)	1,648 (9.69)	19,228	

Table 4 presents the results from a series of multivariable logistic regressions with four IPV exposures. The exposure of having experience of at least one type of IPV was used in Model 1, emotional IPV as an exposure variable was used in Model 2, sexual IPV as an exposure variable was used in Model 3, and physical IPV as an exposure variable was used in Model 4, respectively. The results revealed that the Model 1 with at least one type of IPV (aOR= 1.32, 95% CI = 1.08–1.62) and Model 2 with emotional IPV (aOR = 1.35, 95% CI = 1.09–1.67) showed a significant association with CC screening.

**Table 4.** Results from Multivariable Logistic Regressions: Ever Had Cervical Cancer Screening

	Outcome: Ever had cervical cancer screening			
	Model 1: aOR (95% CI)	Model 2: aOR (95% CI)	Model 3: aOR (95% CI)	Model 4: aOR (95% CI)
Experienced of IPV				
At least one type of intimate partner violence				
No	Reference			
Yes	1.32 (1.08, 1.62)**			
Emotional violence				
No		Reference		
Yes		1.35 (1.09, 1.67)**		
Sexual violence				
No			Reference	
Physical violence			1.47 (0.93, 2.34)	
No				Reference
Yes				1.26 (0.94, 1.67)
Age groups				
15–19	Reference	Reference	Reference	Reference
20–24	1.34 (0.53, 3.39)	1.33 (0.52, 3.37)	1.32 (0.51, 3.37)	1.32 (0.52, 3.37)
25–29	2.47 (1.03, 5.9)*	2.45 (1.02, 5.86)*	2.42 (1.00, 5.83)*	2.41 (1.00, 5.79)*
30–34	3.68 (1.53, 8.83)***	3.66 (1.53, 8.78)**	3.62 (1.49, 8.76)**	3.61 (1.49, 8.72)**
35–39	3.91 (1.64, 9.32)***	3.88 (1.63, 9.26)**	3.81 (1.59, 9.16)**	3.82 (1.59, 9.16)**
40–44	4.71 (1.97, 11.25)***	4.68 (1.96, 11.18)***	4.64 (1.92, 11.18)***	4.63 (1.92, 11.12)**
45–49	6.42 (2.60, 15.45)***	6.38 (2.65, 15.35)***	6.25 (2.57, 15.17)***	6.26 (2.5, 15.16)***
Place of residence				
Urban	Reference	Reference	Reference	Reference
Rural	0.85 (0.69, 1.04)	0.85 (0.69, 1.04)	0.86 (0.71, 1.05)	0.86 (0.70, 1.05)
Education level				
No education	Reference	Reference	Reference	Reference
Primary	7.58 (1.74, 33.02)**	7.63 (1.75, 33.24)**	7.78 (1.78, 33.87)**	7.78 (1.78, 33.86)**
Secondary	10.6 (2.47, 46.28)***	10.7 (2.47, 46.43)**	10.87 (2.51, 47.04)***	10.87 (2.51, 47.05)***
Higher	14.26 (3.28, 61.99)***	14.29 (3.29, 62.10)***	14.40 (3.33, 62.74)***	14.45 (3.32, 62.77)***
Wealth quintile				
Poorest	Reference	Reference	Reference	Reference
Poorer	1.52 (1.12, 2.05)**	1.51 (1.12, 2.05)**	1.53 (1.13, 2.07)**	1.53 (1.12, 2.07)**
Middle	1.98 (1.47, 2.66)***	1.98 (1.47, 2.67)***	1.98 (1.47, 2.67)***	1.98 (1.47, 2.67)***
Richer	2.31 (1.68, 3.18)***	2.31 (1.68, 3.18)***	2.32 (1, 3.18)***	2.32 (1.68, 3.19)***
Richest	3.46 (2.54, 4.72)***	3.44 (2.53, 4.70)***	3.44 (2.52, 4.69)***	3.45 (2.53, 4.72)***
Marital status				
Never in union	Reference	Reference	Reference	Reference
Currently in union/living with a man	1.22 (0.76, 1.92)	1.22 (0.77, 1.93)	1.22 (0.77, 1.93)	1.2 (0.758, 1.91)
Formerly in union/living with a man	0.76 (0.41, 1.42)	0.75 (0.4, 1.40)	0.79 (0.43, 1.49)	0.79 (0.42, 1.47)
Religion				
Other religions	Reference	Reference	Reference	Reference
Roman Catholics	1.14 (0.89, 1.45)	1.14 (0.89, 1.44)	1.14 (0.89, 1.45)	1.13 (0.90, 1.45)
Current employment				
No	Reference	Reference	Reference	Reference
Yes	1.30 (1.08, 1.57)**	1.30 (1.08, 1.57)**	1.30 (1.08, 1.56)**	1.30 (1.08, 1.56)**
Number of lifetime sexual partners				
Only one	Reference	Reference	Reference	Reference
Two to five	1.53 (1.26, 1.84)***	1.53 (1.26, 1.85)***	1.55 (1.29, 1.87)***	1.55 (1.28, 1.87)***
More than five	3.16 (1.00, 9.97)*	3.19 (1.03, 9.94)*	3.31 (1.07, 10.16)*	3.23 (1.03, 10.05)*
Parity				
No child	Reference	Reference	Reference	Reference
Only one	0.99 (0.7, 1.41)	0.99 (0.7, 1.41)	1.01 (0.71, 1.43)	1.01 (0.71, 1.44)
Two to five	1.01 (0.7, 1.46)	1.01 (0.7, 1.46)	1.03 (0.72, 1.49)	1.03 (0.71, 1.49)
More than five	0.72 (0.39, 1.29)	0.72 (0.39, 1.29)	0.73 (0.41, 1.31)	0.73 (0.41, 1.31)
Access to health care				
Not a big problem	Reference	Reference	Reference	Reference
A big problem	1.09 (0.93, 1.20)	1.09 (0.92, 1.28)	1.11 (0.95, 1.31)	1.11 (0.94, 1.31)
_cons	0.00 (0.00, 0.01)***	0.00 (0.00, 0.00)***	0.00 (0.00, 0.01)***	0.00 (0.00, 0.01)***

In all four models, women who are in the older age groups, who have some education level, who are wealthier, who are currently working, and who have had more than one sexual partner were more likely to have CC screening than those who were not. The higher odds ratios were observed as the age gets older. For example, in Model 1, the women aged 45–49 years were 6.42 times more likely to get CC screening than those who were in the 15–19 age groups. The women with a higher education level were more likely to get tested for CC than those who did not have any education. The highest odds ratios were seen among the women with higher education (aOR = 14.26, 95% CI = 3.28–61.99 in Model 1, aOR = 14.29, 95% CI = 3.29–62.10 in Model 2, aOR = 14.40, 95% CI = 3.33–62.74 in Model 3, and aOR = 14.45, 95% CI = 3.32–62.77 in Model 4). Likewise, compared with women in the poorest group, the women in other wealth quintile groups were more likely to undergo CC screening, and the highest odds ratios were seen among the wealthier groups (aOR = 3.46, 95% CI = 2.54-4.72 in Model 1, aOR = 3.44, 95% CI = 2.53–4.70 in Model 2, aOR = 3.44, 95% CI = 2.52–4.69 in Model 3, and aOR = 3.45, 95% CI = 2.53–4.72 in Model 4). In all models, the women who were currently employed were 1.3 times more likely to undergo CC screening than those who did not. Similarly, women who had more than one sexual partner were 1.5 times more likely to get tested for CC than those who had only one partner. We found no significant association between the CC screening and marital status, religion, parity, and access to the nearest health facility. Table 4 presents the details of the multivariable logistic regression results.

## Discussion

The findings from the present study bridge the substantial gaps in CC screening among IPV survivors in the Philippines. To our knowledge, this is the first article to analyze the different types of IPV and the likelihood of CC screening among Filipino women using the most recent nationally representative data of the Philippines. Overall, approximately 9.69% of the study participants had screened for CC. Among the women who experienced IPV, the CC screening rate ranged from 15% (among those who had emotional and physical IPV) to 17% (among those who reported sexual IPVs).

Our findings demonstrate that women who experienced sexual and physical violence were more likely to get tested for CC. However, we did not find the association between CC screening and experiences of sexual and physical violence statistically significant. Our findings regarding insignificant association between CC screening and either sexual or physical IPVs were consistent with a previous study.^38^ Potential reasons could be the limitation of sample size and possible underreporting of IPV, with only 2% of women reporting experiencing sexual violence and 6% reporting experiencing physical violence. In the Philippines, discussing topics related to sex and sexual education has been considered taboo, which further extends to conversations and disclosure of IPV.^44^ This societal perception may hinder IPV victims from accessing health care facilities and receiving early treatment and care.^41^

The study demonstrated that the women experiencing emotional IPV were more likely to screen for CC than those who did not experience such violence and the relationship was significant. A possible explanation for this phenomenon is that women experiencing emotional IPV perceive higher risks of CC, which may prompt them to seek screening. Also, it has been shown that violent intimate partners are more likely to have multiple sexual partners, which may also prompt victims of IPV to seek screening for CC.^35^ Both of these potential explanations need further exploration. A recent study in Sweden conducted in 2019 by Lu and colleagues found that women who reported stress were 33% more likely to die of CC or unspecified uterine cancer compared with those who did not report stress.^45^ One possible explanation for this disparity could be emotion-caused delay in seeking health care, leading to a higher mortality. Therefore, the findings suggest that women with emotional violence may be more susceptible to CC and higher CC-related mortality, therefore subsequent treatment after CC screening is warranted.

The multivariable logistic regression results in the significant association between CC screening and women in the older age groups (aged 20–49 years) who may have been influenced by the CC screening guideline. The Philippines National Guideline-recommended age for CC screening for women is 25–55 years.^15^ This could be one of the reasons that support our finding that women 20 years of age and older were more likely to get tested for CC than those who were from the 15–19 age group. However, our findings also revealed that CC cases are reported among women 19 years of age and younger.^19^ Although the Philippines guideline is proactive in screening women at a younger age compared with the WHO guideline, which recommends CC screening for women 30 years of age and older for women in general, there was no active screening program in the Philippines.^19^ Urgent attention for the active screening program should be taken to achieve the goal of eliminating CC in the Philippines.^31,46^

The findings of the significant association between the CC screening among women with a higher education compared with those who had no education is consistent with a couple of other study's findings.^40,47^ The study conducted in the United States by Coughlin and colleagues (2006) found that women with lower education living in communities where residents have lower education were less likely to undergo CC screening than those living in other communities.^47^ Moreover, the study conducted in South Korea by Chang and colleagues (2017) revealed that the women with more than 12 years of education were more likely to undergo CC screening.^48^ Although the Philippines’ literacy rate is more than 90%, ensuring to reach the women with no education is critical to narrow the health equity gaps. Further research with a focus on individual- and societal-level education could be beneficial to understand the uptake of CC screening in the Philippines.^19^

Our findings also revealed that women who were currently employed were more likely to undergo CC screening than those who were not. Our finding is supported by a couple of previous studies.^49,50^ Fedewa and colleagues (2022)’ study conducted in the United States revealed unemployed individuals had a lower level of CC screening conducted.^49^ Likewise, Gizaw and colleagues (2022) also found that the women who were employed, especially in the governmental organizations, were more likely to undergo CC screening than those who were not.^50^ Although our study did not specifically evaluate the place of employment, such factors may need to be explored further to understand the CC-related screening behavior among women in the Philippines.

Our findings regarding the CC screening and place of residence, marital status, and religion showed no significance although some studies revealed a significant association.^50–53^ Although the result from the present study showed that the women who lived in the rural areas were less likely to uptake CC screening than those living in the urban areas, the association was not significant. This result was contradicted with a previous study conducted by Mpachika-Mfipa and colleagues (2022), in which a significant association was observed between semiurban residents being more likely to be screened for CC than those living in the rural areas.^53^ Likewise, the findings on CC screening and religion (*i.e.,* Roman Catholic, which is the major religion in the Philippines) did not show any significant association in our study, although Mpachika-Mfipa and colleagues (2022) found that Christian women were more likely to undergo CC screening than Islamic women.^53^ These areas could be explored further to understand the religious influences on health service uptake.

Similarly, our findings did not reveal any significant association between CC screening and marital status, although a previous study in the United States found that married women were more likely to be screened for CC than those who never married.^52^ A study conducted in Southwest Ethiopia by Gizaw and colleagues also found that married women were more likely to undergo CC screening than those who were not married.^50^ Likewise, our findings did not show any significant association between CC screening and whether the respondents felt that it is a big problem accessing health care. However, a couple of studies demonstrated the association between CC screening and the perceived barriers to the nearest health facilities.^50,51^ Gizaw and colleagues found that the women who reported “no big problem” to the health facility were more likely to undergo CC screening than their counterparts.^50^ Moreover, a study conducted in India revealed that the barriers regarding the uptake of CC screening include absence of symptoms, limited knowledge about CC screening, being busy with household chores, family problems, and lack of approval from husbands.^51^ Given the sensitive nature of the questions and disclosure of domestic violence issues to strangers in some cultures may contribute to the underreporting of IPVs, especially regarding sexual violence. Nevertheless, our findings suggest that further in-depth research is recommended to further understand the association between IPV and CC screening.

### Limitations

The present study has a few limitations. First, we used cross-sectional data, and therefore the temporality and the causal inferences cannot be established. Second, this study used self-reported data, which could lead to recall and social desirability bias. This study focuses only on screening and therefore did not focus on vaccination and treatment. Finally, this study could not determine the participant’s knowledge and attitude toward CC screening as such data were not included in the survey.

## Conclusion

This study found the overall prevalence of CC screening is low (9.69%) among Filipino women especially with IPV experiences. This finding indicates that initiatives are warranted to increase the coverage of CC screening in this specific population. The study identified factors associated with the CC screening among Filipino women with IPV experiences. Future research and interventions should focus on active screening by making information available and services affordable and accessible for the most vulnerable women. Moreover, immediate attention is needed to reach those experiencing IPV and experiencing health disparities due to their age, socioeconomic, and education status.

## Data Availability

This study has used publicly available, secondary data, which can be accessed on registering at the DHS program website https://dhsprogram.com/data/Using-DataSets-for-Analysis.cfm.

## Abbreviations Used

aOR: Adjusted odds ratio
CC: Cervical cancer
CI: Confidence Interval
HPV: Human papillomavirus
IPV: Intimate partner violence
NDHS: National Demographic and Health Survey
WHO: World Health Organization
